# Tuberculin skin test for the diagnosis of latent tuberculosis during renal replacement therapy in an endemic area: A single center study

**DOI:** 10.4103/0971-4065.70842

**Published:** 2010-07

**Authors:** S. K. Agarwal, S. Gupta, D. Bhowmik, S. Mahajan

**Affiliations:** Department of Nephrology, AIIMS, New Delhi - 110 029, India

**Keywords:** Latent tuberculosis, renal replacement therapy, tuberculin test

## Abstract

Patients on renal replacement therapy (RRT) are at-risk for developing tuberculosis (TB). There is limited information on tuberculin skin test (TST) and its predictability for development of TB. In this prospective cohort study, patients taken for RRT were included. Patients with active TB were excluded. TST was done with 5-tuberculin unit. In addition to TST, age, sex, diabetes as basic disease, number of dialysis and blood transfusion (BT), pre-transplant TB, hepatitis B and C infections and type of immunosuppression were correlated with the development of TB. Of the 200 patients included, TST was positive in 21 and negative in 179. In TST negative group, 20 (11.1%) and in TST positive group 5 (23.8%) patients developed TB. TB free survival in two groups was similar (*P* = 0.08). On multivariate Cox regression analysis, hazard of development of TB by TST was 2.7 [*P* = 0.11, confidence interval (CI) 0.78-9.7]. There was no difference between TST non-responsive and TST negative patients (*P* = 0.18). Sensitivity and specificity of TST for predicting TB was only 20 and 9%, respectively. Our study shows that TST in patients on dialysis is an insensitive and nonspecific test to predict development of active TB.

## Introduction

Screening patients at high risk for development of tuberculosis (TB) and use of therapy for latent tuberculosis (LTB) infection to prevent the development of active TB is a well-accepted strategy for TB control. Chronic kidney disease (CKD) patients on maintenance hemodialysis (MHD) are a high-risk group for TB.[1–5] These patients are approximately 10 times more likely to get active TB than the general population. Part of the increased risk of TB is caused by progression from LTB to active disease due to impaired immunity associated with CKD.[6] However, routine tuberculin skin testing (TST), also called Mantoux test (MxT), is mostly not performed in these patients, partly because of high rates of anergy among MHD patients.[2,7] Further, in conditions which alter the cutaneous response to TST, like CKD, significance of TST in the diagnosis of LTB is controversial. There is limited information on TST and LTB in MHD patients from India as well as from the other parts of the world. As there is no gold standard with which TST can be compared for the diagnosis of LTB, the best approach is to follow these patients for the development of TB at a later stage and take disease development as gold standard. This study was conducted to find out the significance of TST for the diagnosis of LTB in patients on renal replacement therapy (RRT).

## Materials and Methods

### Design and setting

This is a prospective cohort study conducted at the Department of Nephrology, All India Institute of Medical Sciences, New Delhi.

### Inclusion criteria

- Patients of end stage kidney disease (ESKD) taken for MHD followed by renal transplant (RT) at our hospital, were included in the study. The exclusion criteria are the following:

### Exclusion criteria

- Patients having active TB at the time of inclusion in the study
- All pediatric patients as these patients before and after transplant were followed in the Department of Pediatrics
- Patients who had been given TB prophylaxis in various trial done at our center during the study period

All the patients who were accepted for MHD and RT program were the subjects for the study. All the patients were HIV negative. Patients with active TB were excluded. All patients were subjected to TST following standard method.[8] In short, the test was carried out by intracutaneous inoculation of 0.1 ml tuberculin solution (Span Diagnostic Ltd., Surat, India) containing 5-tuberculin unit (TU) into the volar surface of forearm. Tuberculin was diluted with a special buffer containing Tween-80 as a stabilizer. Source material had been calibrated against batch RT-23 manufactured by Statens Serum Institute, Denmark. The resulting induration was measured 48-72 hours after the test. An induration of 10 mm or more was considered positive for the diagnosis of LTB. All these patients were followed during MHD period as well as after RT. Follow-up during MHD was for a minimum twice a week at the time of dialysis or as and when required. Following RT, 0 follow-up was done weekly for the first 3 months, once in 2 weeks for the next 3 months and monthly during next 6 months. A thorough physical examination was done during each visit. Any onset of TB was confirmed by microbiological/histologic testing. Patients having fever without any localizing sign/investigation were, as per our protocol, subjected to ultrasound abdomen for any retroperitoneal lymph node enlargement, computerized tomography (CT) scan of the chest for mediastinal lymph node enlargement and bone marrow examination for evidence of TB. If all these three tests were found normal, only then a trial of anti-tubercular therapy (ATT) was given and the response to therapy was taken as evidence of TB.

All patients were on calcinurin inhibitor [Cyclosporin (CsA) or Tacrolimus (TAC)] based triple immunosuppressant regimen which was described in detail in an earlier report.[9] Our basic immunosuppression protocol in these patients had been steroid, CsA and azathioprin. We could used tacrolimus and mycophenolate mofetil only in those patients who could afford these drugs.

In addition to results of TST, age of patient, male sex, diabetes as cause of ESKD, number of hemodialysis and BT, pre-RT tuberculosis, hepatitis B and hepatitis C viral infections and type of immunosuppression were correlated with the development of TB.

Data were recorded on a pre-designed proforma and managed on an excel spread sheet. All the entries were checked for an error. Categorical variables were summarized by frequency and their percentage. Quantitative variables were assessed for their approximate normal distribution. The variables following approximate normal distribution, their mean values, were compared using Student’s *t*-test. For the non-normal variables, Wilcoxon’s rank sum test was used to compare median values in different groups. To evaluate the effect of various variables, Cox regression analysis was done. Kaplan-Meier method was used to plot TB free survival curve between two groups. The overall TB free survival curves in the two groups were compared using log rank test. Stata 10 (Intercooled version) was used for statistical analysis. A *P* value of less than 0.05 was considered to indicate statistical significance.

An informed consent was obtained from the subjects and the protocol was approved by institute’s ethic committee.

## Results

**Figure 1:**
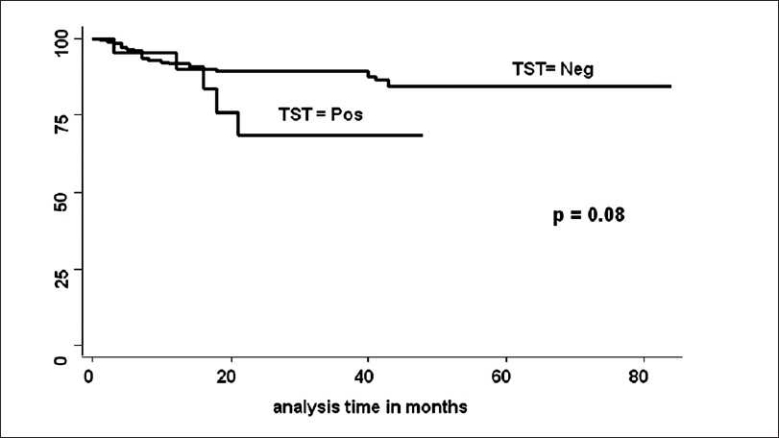
Tuberculosis free survival by TST

Between May 2000 to May 2006, 554 patients got MHD followed by RT at our hospital. Pediatric patients (23), patients having active TB (57), patients getting TB prophylaxis in clinical trials done at our institute (212) and patients in whom TST could not be done before RT (62), were excluded from the study. Remaining 200 patients were included for the analysis for determining TST for LTB, TST was positive in 21 and negative in 179. Details of the patients are shown in Table 1. In TST negative group 20 (11.1%) and in TST positive group 5 (23.8%) patients developed TB following RT. None of the patients developed TB before RT while he/she was on maintenance dialysis. This is also because of the fact that majority of patients remained on maintenance dialysis for just few weeks before RT was done. Patients who had TB before TST was done were analyzed as having history of TB. Of the 20 cases in TST negative group, who developed post-RT tuberculosis the site of lesion were 1 disseminated TB (bone marrow acid fast bacilli AFB positive), 3 exudative pleural effusion, 5 pulmonary TB (4 bronchoalveolar levage positive), 2 tubercular meningitis, 3 miliary TB, 5 pyrexia of unknown origin (PUO) and one skin TB (AFB positive) were found. In the TST positive group, two had miliary TB, two had pulmonary TB (one bronchoalveolar levage AFB and one sputum AFB positive) and one case had PUO. There was no death attributed directly to TB. However, in one patient it was associated with cytomegalovirus (CMV) infection. In univariate analysis, the follow-up was longer in TST negative group. The number of acute rejection (AR) and new onset diabetes mellitus after transplantation (NODAT) before development of TB were significantly more in TST negative group. On the contrary, CMV disease before development of TB was more in TST positive group. On multivariate Cox regression analysis, while adjusting follow-up period, MMF, tacrolimus, induction therapy, AR, NODAT and CMV before development of TB, hazard of positive TST for development of post-RT TB was 2.7 [*P* = 0.11, confidence interval (CI) 0.78-9.7]. TB free survival estimates by TST positivity were not significant [Figure 1](log rank test, *P* = 0.08).

**Table 1:** Clinical details between TST positive and negative groups

	TST negative	TST positive	*P* value
No. of cases	179	21	
Mean age (years)	34.24 ± 10.9	38.3 ± 12.2	0.6
Mean ± SD (range)	(14-60)	(19-60)	
% Males	152 (84.9%)	17 (80.9%)	0.6
Diabetes as cause of ESKD	16 (9%)	4 (19%)	0.14
Follow-up (months)	33.4 ± 21.9	24.0 ± 13.4	0.05
Mean ± SD (range)	(1-84)	(2-48)	
No of HD	57.5 ± 49.9	51.9 ± 27.6	0.61
Mean ± SD (range)	(1-414)	(0-100)	
No of BT	4.5 ± 6.0	4.1 ± 5.1	0.76
Mean ± SD (range)	(0-53)	(0-20)	
Pre-RT TB	14 (7.8%)	1 (4.8%)	0.62
HBV infection	7 (3.9%)	Nil	0.35
HCV infection	34 (18.9%)	4 (19%)	0.9
Immunosuppression			
Tacrolimus	37 (20.7%)	7 (33.3%)	0.16
MMF	34 (19%)	5 (23.8%)	0.6
Induction	15 (8.4%)	4 (19%)	0.09
No. of AR	3 (15%)	Nil	0.05
Before post-RT TB no. of PTDM	5 (25%)	Nil	0.009
Before post-RT TB no. of CMV	1 (5%)	1 (20%)	0.008
Before post-RT TB no. of cases developing TB	20 (11.1%)	5 (23.8%)	0.08
Mean time since RT (months)	14 ± 6.9	14.8 ± 15.0	0.67
Mean ± SD (range)	(3-21)	(1-47)	

TST = tuberculin skin test; ESKD = end stage kidney disease; HD = hemodialysis; BT = blood transfusion; RT = renal transplantation; TB = tuberculosis; AR = acute rejection; PTDM = post transplant diabetes mellitus; CMV = cytomegalovirus infection; HBV = hepatitis B virus; HCV = hepatitis C virus; <-before

Of the 179 patients who were negative for TST, 156 (78%) did not show any reaction to TST. TB free survival estimates by TST results also had no significant difference in survival between TST negative vs. anergy on TST test (log rank test, *P* = 0.18). On multivariate Cox regression analysis, while adjusting follow-up, AR and NODAT before development of TB, there was no significant risk of developing TB in patients who were positive for TST as compared to TST negative patients.

## Discussion

TB prevention and control includes three strategies: a) identifying and treating persons who have active TB, b) screening persons who have had contact with TB patients to determine whether they are infected with *Mycobacterium tuberculosis* or have active TB, and providing appropriate treatment and c) screening and treatment of populations at high risk for TB infection to prevent progression to active TB.[10] The practice of screening low-risk groups is not recommended. Patients on MHD are a high-risk group and deserve screening.[11] The American thoracic Society and Centre for Disease Control[11] it is clearly documented that patients of CKD on MHD need tuberculin testing for diagnosis of LTB. It is also made clear that in countries like India where bacilli Calmette-Guerin BCG is used for vaccination for tuberculosis, there is no test that differentiates between BCG effect vs. LTB. Thus, till a new test is developed, TST is the only test that can be used for diagnosis of LTB in this clinical setting. We at our own institution had previously published that isoniazid (INH) prophylaxis in these patients can prevent active TB.[11–13] However, in an endemic area, giving prophylaxis to every patient going for MHD followed by RT may not be justified. Thus, we need to detect patients at significant risk of developing TB. In this regard, TST till now should be the accepted test for the diagnosis and management of LTB.

There are a few reports on TST in relation to patients on RRT, in the literature.[14–26] Major features of these studies have been shown in Table 2. The prevalence of TST positivity ranged from 9% to 55%. However, no definite factors could be attributed to suggest the high prevalence in some studies.[16,17,19,24,25] In some studies, it has been shown that a repeat booster testing after few days of first skin test had improved the positivity of TST test.[16,17,20,24] But its relevance in terms of treatment or future development of active disease has not been evaluated. Some of the factors attributed in different studies increasing TST positivity are male sex,[20,25] elderly patients,[23] past history of TB[17] and better nutrition.[19] In this study nearly 80% patients were males and only 5-8% patients had history of past TB.

**Table 2:** Details of studies on TST in RRT

Year	First author	Ref.	Patient group	No.	% TST	% anergy	Specific comment on study
1998	Smirnoff	[12]	MHD		19	40	—
1998	Woeltje	[13]	MHD	307	9	32	INH prophylaxis to 12% TST+ patients
2003	Akcay	[14]	MHD	53	35.8	—	Booster TST improved positivity
2003	Poduval	[15]	MHD	118	35	52.5	50% got INH prophylaxis
							Booster improved TST positivity
2004	Wauters	[16]	MHD	224	14.7	—	Weak association with past + TST and TB history
							Better nutrition has association with TST
2005	Shanker	[17]	MHD	108	44	44	Four patients had TB after 79 RT patients
2005	Dogan	[18]	MHD	124	11.3	—	Booster improved positivity to 23.4%
							Healthy donor, controls also taken
							Donor had 11.2% TST positivity
							TST positivity was in all males
2006	Khosroshahi	[19]	MHD+	308	33.6	—	Age had relation with positivity
							Also had CKD, CAPD and RT patients
							TST positivity taken as >5 mm
2006	Basoglu	[20]	MHD +	44	50	—	No correlation with T-cell dysfunction
2006	Kantarci	[21]	MHD	164	42	43.3	Elderly had high TST positivity
2006	Cengiz	[22]	MHD	106	37.7	36.8	Booster increased TST positivity
							Booster increased TST to 68.8%
2007	Habesoglu	[23]	MHD	187	55.1	—	Association with past TST and males
2007	Passalent	[24]	MHD	203	12.8	—	T-spot (35.5%) and TST comparison

TST = tuberculin skin test; Ref. = reference; MHD = maintenance hemodialysis; MHD+ = maintenance hemodialysis and others; TB = tuberculosis; RT = renal transplant; CKD = chronic kidney disease; CAPD = continuous ambulatory peritoneal dialysis

The main aim of this study was to see the effect of TST on future development of active TB. There is only one study from Indian subcontinent,[19] which can be compared with the present study. It included 108 patients and of them only 79 patients were followed-up after RT. The maximum follow-up period was 30 months, while in our study we had longest follow-up up to 8 years. Though post-RT TB is more common in first year, we have shown that cumulative incidence of post-RT TB goes on increasing even after the first year (unpublished data). However, even with a large number of patients and longer follow-up, we did not find any significant difference in the risk of development of TB in TST positive as compared to TST negative patients. As per our results, sensitivity of TST for predicting post-RT TB is 20% and specificity is 9% only.

T-cell dysfunction in CKD has been suggested as one of the reasons for TST-hypo and unresponsiveness. However, Basoglu *et al*,[22] could not demonstrate T-cell dysfunction and its association with TST response. Further, Passalent *et al*,[26] while comparing T-spot test with TST in patients on MHD, demonstrated that T-spot gave 35% positivity as compared to TST, which showed positive response in 12.8% patients.

Discovery of the role of T lymphocytes and interferon-γ assay (IFN-γ) in the immune process has led to the development of an *in vitro* assay for cell-mediated immune reactions to *M. tuberculosis*. [27] The IFN-γ assay is a whole-blood test for diagnosing LTB. If a patient is infected with *M. tuberculosis*, the lymphocytes recognize the tuberculin and release IFN-γ in response. The test is found to be moderately concordant with TST.[28] TST and IFN-γ assay do not measure the same component of the immunologic response and are not interchangeable. Streeton *et al*,[29] in their study using IFN-γ assay test showed a specificity and sensitivity of 98 and 90%, respectively. Converse *et al*,[30] studied the suitability of IFN-γ assay in high-risk adults with intravenous drug abuse. However, there are no adequate published data of this test in CKD patients on RRT.

Our study has some limitations. Firstly, we have not used other antigens to do skin test for demonstration of anergy in these patients. Secondly, in some of the patients the diagnosis of TB was made on the basis of response to ATT and one can question the diagnosis in these patients. However, once the same criteria have been followed in both TST positive and negative groups, this should not have any effect while comparing two groups.

To conclude, our study shows that TST in patients on maintenance hemodialysis is an insensitive and nonspecific test to make a diagnosis of LTB infection and this should not be taken as the criterion to prescribe prophylaxis of TB in these patients.
